# Hypertensive encephalopathy and cerebral infarction

**DOI:** 10.1186/2193-1801-3-741

**Published:** 2014-12-16

**Authors:** Bengt Edvardsson

**Affiliations:** Department of Clinical Sciences, Lund, Neurology, Skane University Hospital, Lund University, S-221 85 Lund, Sweden

**Keywords:** Hypertensive encephalopathy, Posterior reversible encephalopathy syndrome, Magnetic resonance imaging, Cerebral infarction, Ischemia

## Abstract

**Introduction:**

Hypertensive encephalopathy is one cause of posterior reversible encephalopathy syndrome. Hypertensive encephalopathy and cerebral infarction have only been reported in a few individual case reports.

**Case description:**

A 51-year-old woman presented with hypertensive encephalopathy. T2-weighted images from magnetic resonance imaging showed hyperintense lesions in both occipital and parietal lobes. Diffusion-weighted imaging showed that this represented cytotoxic oedema and perfusion magnetic resonance imaging revealed reduced blood volume and flow. The magnetic resonance imaging was repeated 5 months later and subtotal regression of theT2-hyperintensity had occurred. However, small bilateral infarcts were seen on T1-weighted images. Perfusion magnetic resonance imaging presented reduced blood volume and flow on the right side.

**Discussion and evaluation:**

The patient in this report had posterior reversible encephalopathy syndrome caused by hypertensive encephalopathy. Magnetic resonance imaging of the brain showed bilateral cytotoxic oedema that partially resolved and resulted in small infarcts. The imaging findings are compatible with posterior reversible encephalopathy syndrome with subtotal resolution and infarct evolution.

**Conclusion:**

The case report suggests that the presence of hypertensive encephalopathy and posterior reversible encephalopathy syndrome should alert clinicians and lead to prompt treatment in order to prevent cerebral damage.

## Background

In 1996, (Hinchey et al. 1996) first described “reversible posterior leukoencephalopathy syndrome” (RPLS) with different symptoms and imaging findings in connection with hypertensive emergency. Posterior reversible encephalopathy syndrome (PRES) is now commonly used to describe the syndrome (Casey et al. 2000; Covarrubias et al. 2002). Hypertensive encephalopathy (HE) is one cause of PRES. The clinical presentation is diverse (Hinchey et al. 1996), which is why the diagnosis can be misjudged. Symptoms include headache, seizures, vomiting, change in mental status, and cortical visual disturbances. Differential diagnoses include central venous thrombosis, encephalitis, demyelinating disorders, stroke and brain tumour. Apart from HE, PRES has also been associated with pre-eclampsia/eclampsia (Sibai 1996), thrombotic thrombocytopenic purpura/haemolytic uraemic syndrome (Bakshi et al. 1999), cyclosporine A treatment (Gijtenbeek et al. 1999), tacrolismus, interferon alpha-2 and many other conditions.

Computed tomography (CT) typically shows hypodense foci in both parietal and occipital lobes. MRI shows hyperintense lesions on T2-weighted images, in the same regions, reflecting cortical/subcortical oedema with predilection for the posterior circulation (Sanders et al. 1991). Diffusion weighted imaging (DWI) usually shows increased diffusion consistent with vasogenic oedema (Schaefer et al. 1997; Schwartz et al. 1998; Ahn et al. 2004).

I present a patient who presented with HE and subsequently developed PRES with cytotoxic oedema with subtotal regression and development of small infarctions. I postulate that her HE/PRES was related to severe hypertension with fluctuating blood pressure. PRES caused by hypertensive encephalopathy that ends in cerebral infarction is seldom described in individual reports.

## Case description

**Figure 1:**
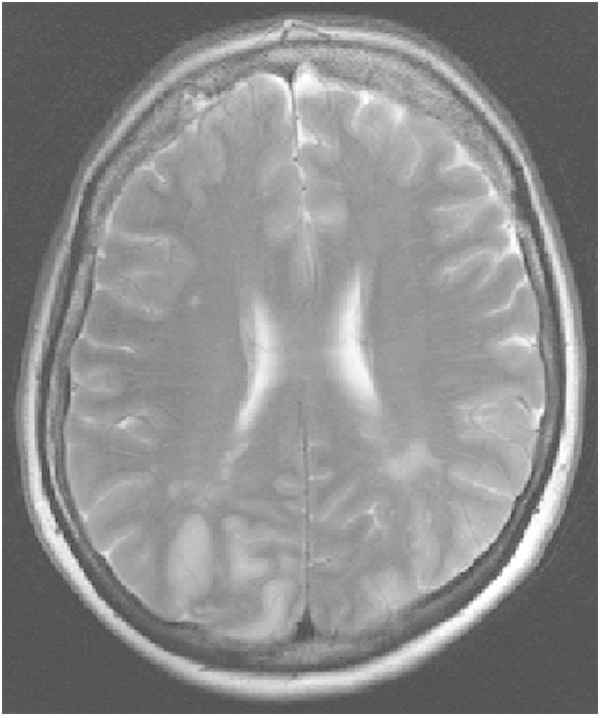
**Primary MRI, T2-weighted image.**

**Figure 2:**
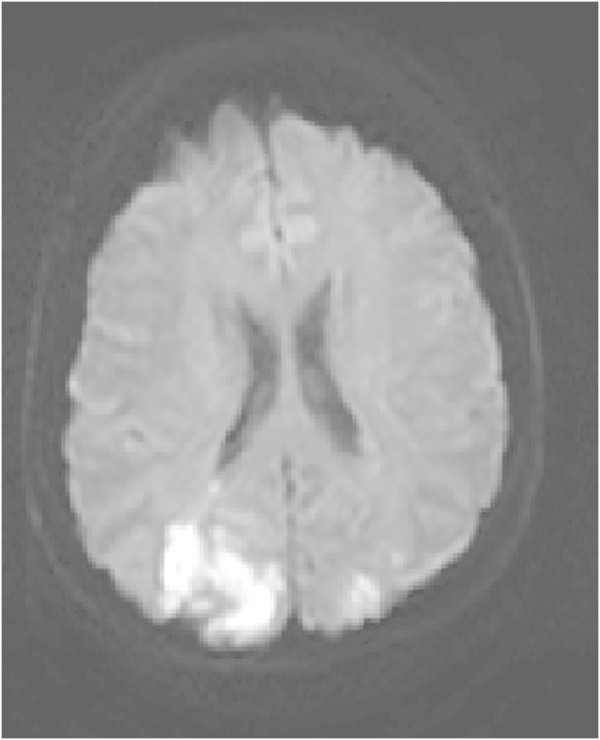
**Primary DWI.**

**Figure 3:**
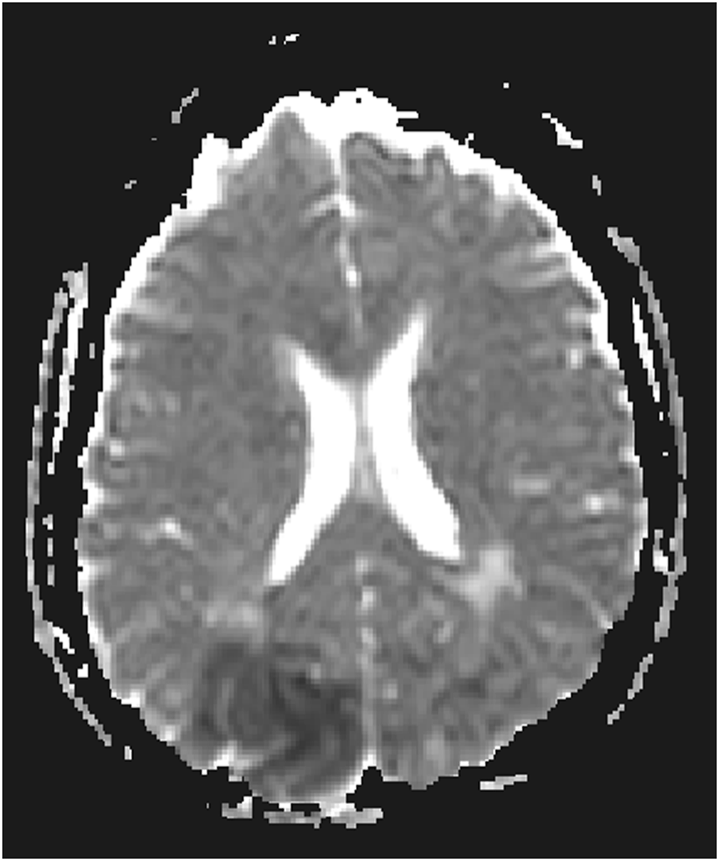
**Primary ADC map.**

**Figure 4:**
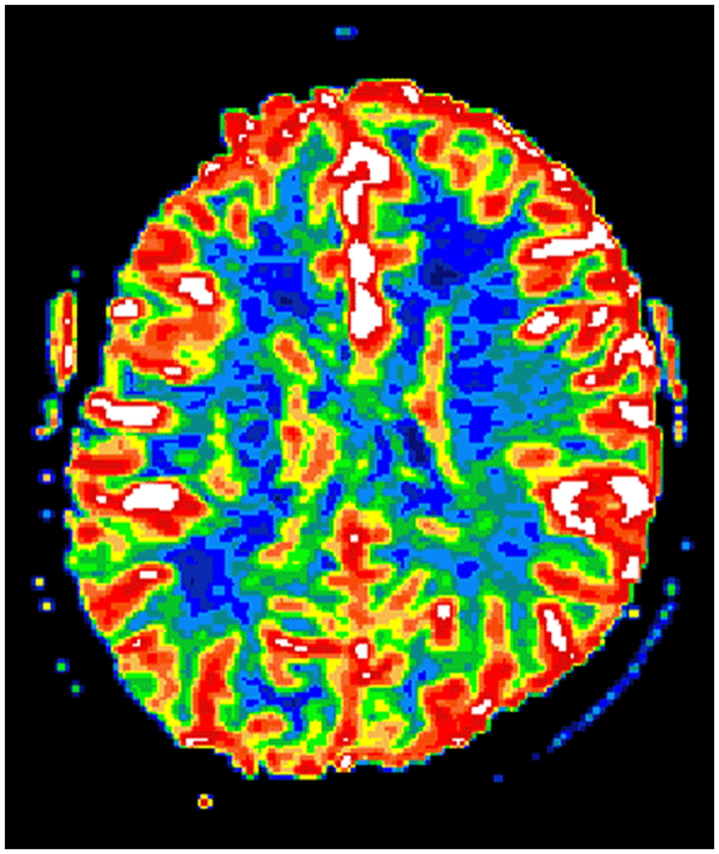
**Primary PWI.**

**Figure 5:**
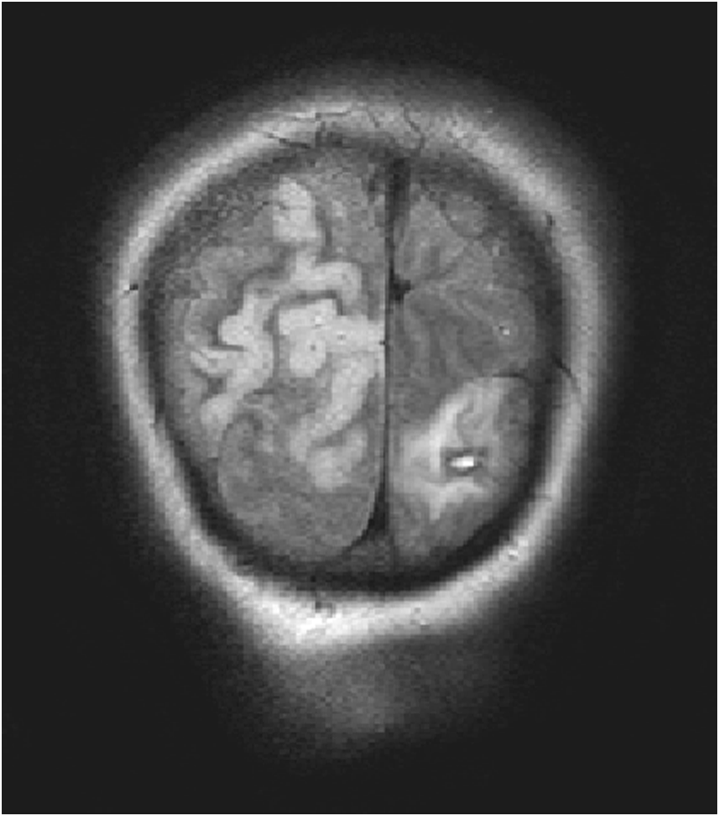
**Primary coronal T2-weighted FLAIR image.**

A 51-year-old, previously healthy, woman experienced headache. Blood pressure had been monitored about one year earlier and she had been normotensive. At the time of admission she had hypertension with a blood pressure of 220/110 mmHg. CT of the brain was normal and cerebrospinal fluid examination was also normal. A physical examination showed nothing remarkable except for high blood pressure. The patient was discharged without further investigation or treatment. Some days later, she noticed a tingling sensation in the right part of her body. This was probably due to a seizure, and she was re-admitted. She had persistent severe headache on the days that followed, intermittently requiring morphine. Nausea and vomiting occurred frequently, decreased alertness was observed, and blurred vision gradually developed. Treatment with phenytoin and levetiracetam intravenously proved to be effective and the seizures stopped. Her blood pressure fluctuated, reaching 230/110 mmHg, but a diagnosis of hypertension was confirmed and treatment started. The hypertension was treated with labetalol and enalaprilat intravenously. After three days, the patient suddenly developed a left-sided hemiparesis and a left-sided hemianopia. The blood pressure at the time of symptom onset was reduced to 190/100 mmHg. MRI, T2-weighted images showed hyperintensity in both occipital and parietal lobes (Figure 1). DWI (Figure 2) reveals rather extensive areas with high signal intensity with low signal on the ADC map (Figure 3) reflecting cytotoxic oedema in both occipital and parietal lobes. PWI showed slightly reduced blood flow on the right side (Figure 4). Coronal T2-weighted FLAIR image shows the bilateral high signal intensity oedema. There was a small hemorrhagic component in the left occipital lobe and adjacent to it there was a small area with high signal (Figure 5). Magnetic resonance angiography (MRA) displayed no signs of vasospasm or intracranial/extracranial stenosis. Complete blood picture, and blood biochemistry were essentially normal. An electroencephalogram (EEG) was ordered and showed diffuse slowing indicating an encephalopathy. Echocardiography was essentially normal. Repeated electrocardiograms were normal. Her blood pressure improved and was 150/100 when the patient was discharged. Her symptoms diminished but she had residual hemianopia.

**Figure 6:**
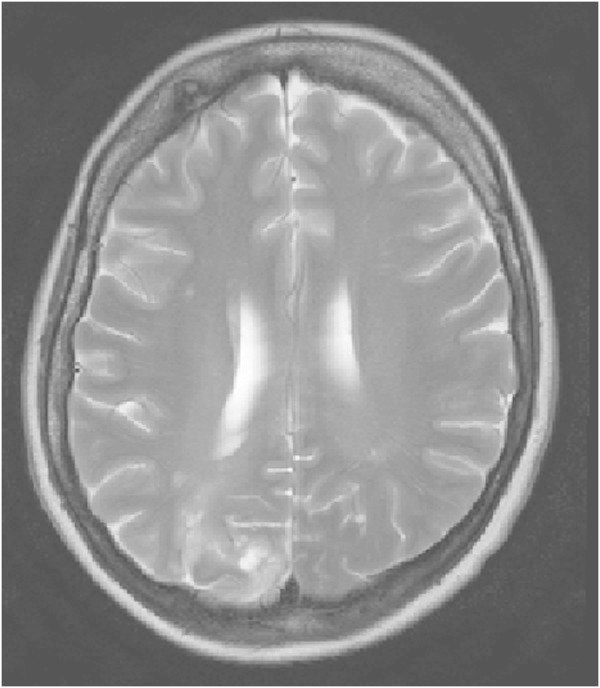
**MRI after 5 months, T2-weighted image.**

**Figure 7:**
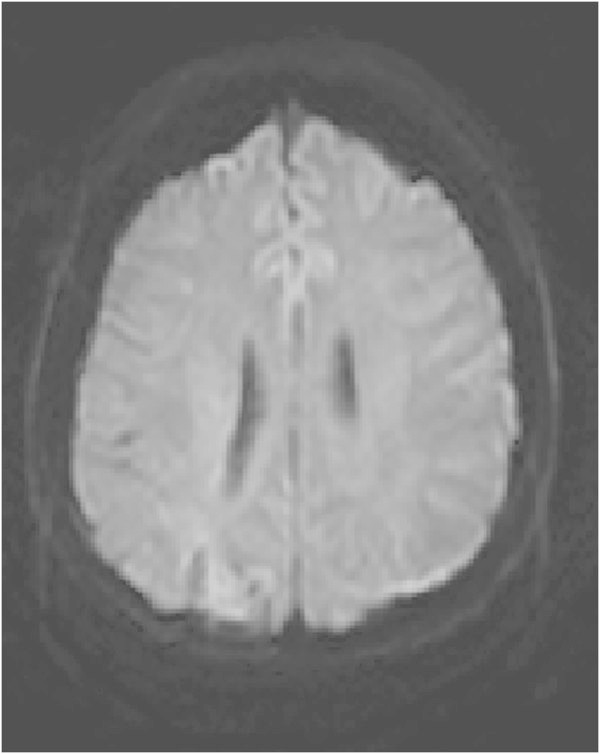
**DWI after 5 months.**

**Figure 8:**
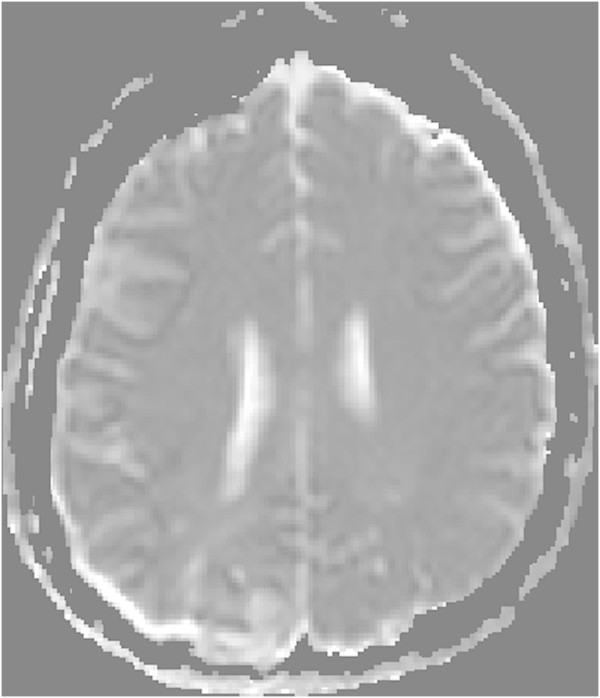
**ADC map after 5 months.**

**Figure 9:**
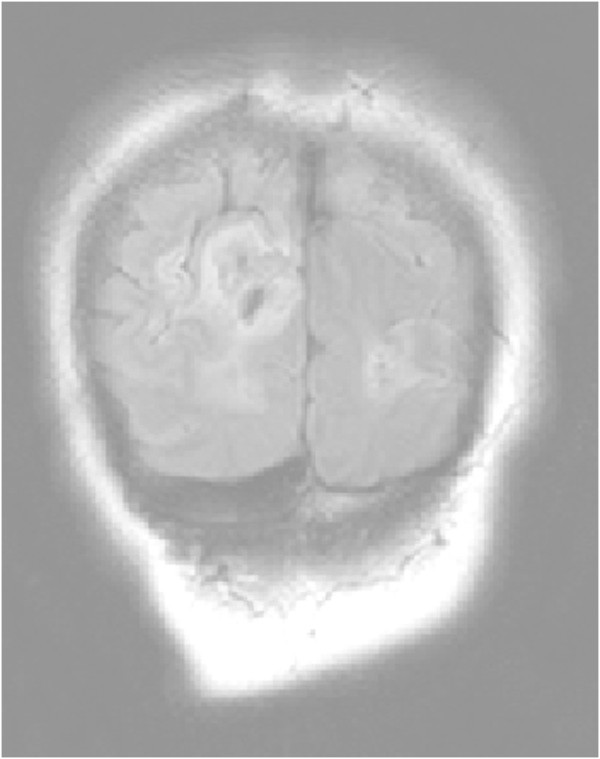
**Coronal FLAIR after 5 months.**

**Figure 10:**
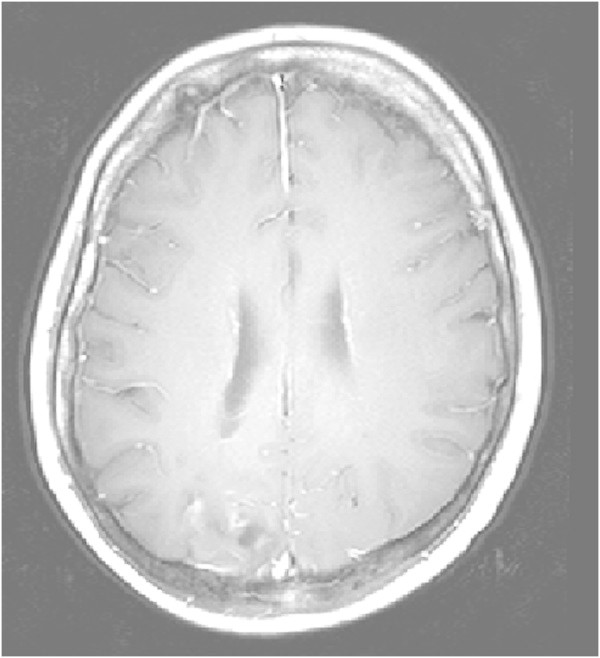
**MRI after 5 months, transverse T1-weighted image after contrast injection.**

On follow-up MRI 5 months later, the T2-weighted images showed subtotal regression of the hyperintense lesions in both occipital lobes, and small areas consistent with infarctions had developed bilaterally (Figures 6, 7, 8, 9 and 10). An extensive clinical work-up has not provided any explanation for the hypertensive crisis that caused her encephalopathy. All secondary causes as renal artery stenosis, renal disease, hyperthyroidism, functioning adrenal tumours, coarctation of the aorta and obstructive sleep apnoea were ruled out. A diagnosis of essential hypertension was established. The patient is currently asymptomatic except for residual hemianopia and her blood pressure varies within the normal range. She is continuing with antihypertensive treatment.

## Discussion

The patient in this report had PRES caused by hypertensive encephalopathy. MRI of the brain showed bilateral cytotoxic oedema that partially resolved and resulted in small infarcts.

The pathophysiology of PRES is not fully understood, but there are two main theories. One postulates that severe hypertension breaks cerebral autoregulation, leading to cerebral vasodilatation/hyperperfusion and subsequently to vasogenic cerebral oedema (Hinchey et al. 1996). The hyperperfusion hypothesis seems to be the most applicable in this case of PRES. The alternative hypothesis postulates endothelial dysfunction caused by circulating toxins such as immunosuppressive drugs, sepsis and cytotoxic agents leading to cerebral vasoconstriction/hypoperfusion and subsequent blood–brain barrier dysfunction and secondary vasogenic oedema (Legriel et al. 2011).

The hallmark of PRES is involvement of the posterior part of the cerebral hemispheres. The imaging modality of choice is MRI, typically revealing vasogenic oedema in the subcortical white matter, often also extending into the cortical grey matter. The good outcome that the term PRES implies is not always achieved: fatal and adverse outcome was seen in up to 26% of cases in one report (Covarrubias et al. 2002). In another article, (Li et al. 2012) reported permanent neurologic deficits in 24% of cases in a retrospective review of RPLS. Cerebral damage can be prevented by immediate treatment of PRES, when it is diagnosed as the underlying mechanism (Ay et al. 1998; Mukherjee and Mc Kinstry 2001).

Studies of PRES usually show diffuse MRI signal changes with increased diffusion, consistent with vasogenic oedema, but cytotoxic oedema has also been reported. Most often cytotoxic oedema was found in a single or few patients and in small areas within lesions predominantly representing vasogenic oedema (Covarrubias et al. 2002; Mukherjee and Mc Kinstry 2001: Seneviratne et al. 2005).

HE/PRES is usually reversible by control of blood pressure or removal of the underlying cause (Mirza 2006). Consequently, case reports of HE and PRES associated with cerebral infarction are uncommon. (Liang et al. 2013) described a patient with an isolated brainstem variant of PRES complicated with ischemic stroke. Severe hypertension was noticed. Hypertensive brainstem encephalopathy is earlier reported and it is usually reversible. (Mak et al. 2004) reported two patients with posterior circulation ischemic strokes associated with HE and blood pressure lowering. However, there are more reports demonstrating resolution of ischemic changes in HE/PRES. The resolution of changes seen in these cases is probably due to urgent treatment of the underlying cause (Casey et al. 2004).

The patient described in the present report differs from the typical patient. Several imaging findings implicated an unfavourable outcome. She had early changes with extensive T2 abnormalities, indicating an adverse outcome (Covarrubias et al. 2002). Focal parenchymal haemorrhages most likely predict permanent clinical symptoms (Hefzy et al. 2009). Massive oedema was seen which could impair the microcirculation and lead to ischaemia (Ay et al. 1998). Restricted diffusion, i.e. cytotoxic oedema, is usually indicative of progress to cerebral infarction, as happened with our patient (Casey et al. 2000; Covarrubias et al. 2002; Ay et al. 1998). However, large portions of the areas with cytotoxic oedema were reversible. Perfusion was reduced, which can be an ominous sign (Sundgren et al. 2002; Ay et al. 1998). The reversibility of PRES is not spontaneous. A delay in diagnosis and treatment may lead to permanent damage (Casey et al. 2000; Covarrubias et al. 2002). Ischemia is a known complication of PRES occurring characteristically in the posterior part of the brain (Covarrubias et al. 2002).

The currently existing definition of hypertensive emergency is a blood pressure higher than 180/120 mmHg. The blood pressure must also be associated with end organ dysfunction as HE (Papadopoulos et al. 2010). In this case, the patient was previously healthy with no known hypertension; this is probably the reason for her sensitivity to increasing blood pressure and development of HE. Interestingly, patients without previous hypertension are more likely to develop acute HE even when the blood pressure does not reach values greater than 180/120 mmHg, because of no protective secondary changes in end-organs. Such changes are common in patients with chronic hypertension (Skinhoj and Strandgaard 1973). The fluctuating nature of the blood pressure can also contribute to the development of HE (de Seze et al. 2000).

## Conclusions

I present a patient who presented with HE and subsequently developed PRES with cytotoxic oedema with subtotal regression and development of small infarctions. The clinician should be vigilant and aware of this syndrome and act promptly when a patient presents with such symptoms in order to prevent cerebral damage. HE/PRES should be included on the list of uncommon causes of stroke.

The principal author takes full responsibility for the data presented in this study, analysis of the data, conclusions, and conduct of the research. The principal author had full access to those data and has maintained the right to publish any and all data independent of any third party.

Concerning approval of human studies by the appropriate ethics committee and therefore performed in accordance with the ethical standards laid down in the 1964 Declaration of Helsinki: In this case this is not appreciable.

## Consent

Informed consent from the patient for the case report to be published Yes.

## References

[CR1] Ahn KJ, You WJ, Jeong SL, Lee JW, Kim BS, Lee JH, Yang DW, Son YM, Hahn ST (2004). Atypical manifestations of reversible posterior leukoencephalopathy syndrome: findings on diffusion imaging and ADC mapping. Neuroradiology.

[CR2] Ay H, Buonanno FS, Schaefer PW, Le DA, Wang B, Gonzalez RG, Koroshetz WJ (1998). Posterior leukoencephalopathy without severe hypertension: utility of diffusion weighted MRI. Neurology.

[CR3] Bakshi R, Shaikh ZA, Bates VE, Kinkel PR (1999). Thrombotic thrombocytopenic purpura. Brain CT and MR findings in 12 patients. Neurology.

[CR4] Casey SO, Sampaio RC, Michel E, Truwit CL (2000). Posterior reversible encephalopathy syndrome: utility of fluid-attenuated inversion recovery MR imaging in the detection of cortical and subcortical lesions. AJNR Am J Neuroradiol.

[CR5] Casey SO, McKinney A, Teksam M, Liu H, Truwit CL (2004). CT perfusion imaging in the management of posterior reversible encephalopathy. Neuroradiology.

[CR6] Covarrubias DJ, Luetmer PH, Campeau NG (2002). Posterior reversible encephalopathy syndrome: prognostic utility of quantitative diffusion-weighted MR images. AJNR Am J Neuroradiol.

[CR7] de Seze J, Mastain B, Stojkovic T, Ferriby D, Pruvo JP, Destee A, Vermersch P (2000). Un usual MRI findings of brainstem in arterial hypertension. AJNR Am J Neuroradiol.

[CR8] Gijtenbeek JMM, Van den Bent MJ, Vecht CJ (1999). Cyclosporine neurotoxicity: a review. J Neurol.

[CR9] Hefzy HM, Bartynski WS, Boardman JF, Lacomis D (2009). Hemorrhage in posterior reversible encephalopathy syndrome: imaging and clinical features. AJNR Am J Neuroradiol.

[CR10] Hinchey J, Chaves C, Appignani B, Breen J, Pao L, Wang A, Pessin MS, Lamy C, Mas JL, Caplan LR (1996). A reversible posterior leukoencephalopathy syndrome. N Engl J Med.

[CR11] Legriel S, Pico F, Azoulay E, Vincent JL (2011). Understanding posterior reversible encephalopathy syndrome. Annual Update in Intensive Care and Emergency Medicine, vol 1.

[CR12] Li Y, Gor D, Walicki D, Jenny D, Jones D, Barbour P, Castaldo J (2012). Spectrum and potential pathogenesis of reversible posterior leukoencephalopathy syndrome. J Stroke Cerebrovasc Dis.

[CR13] Liang H, Li D, Xu Z, Luo B (2013). Isolated pons variant of posterior reversible encephalopathy syndrome complicated with ischemic stroke in a young patient. Neurol Sci.

[CR14] Mak W, Chan KH, Cheung RT, Ho SL (2004). Hypertensive encephalopathy: BP lowering complicated by posterior circulation ischemic stroke. Neurology.

[CR15] Mirza A (2006). Posterior reversible encephalopathy syndrome: a variant of hypertensive encephalopathy. J Clin Neurosci.

[CR16] Mukherjee P, Mc Kinstry RC (2001). Reversible posterior leukoencephalopathy syndrome: evaluation with diffusion tensor MR imaging. Radiology.

[CR17] Papadopoulos DP, Mourouzis I, Thomopoulos C, Makris T, Papademetriou V (2010). Hypertension crisis. Blood Press.

[CR18] Sanders T, Clayman D, Sanchez-Ramos L, Vines F, Russo L (1991). Brain in eclampsia: MR imaging with clinical correlation. Radiology.

[CR19] Schaefer PW, Buonanno FS, Gonzalez RG, Schwamm LH (1997). Diffusion-weighted imaging discriminates between cytotoxic and vasogenic edema in a patient with eclampsia. Stroke.

[CR20] Schwartz RB, Mulkern RV, Gudbjartsson H, Jolesz F (1998). Diffusion-weighted MR imaging in hypertensive encephalopathy: clues to pathogenesis. AJNR Am J Neuroradiol.

[CR21] Seneviratne J, Brotchie P, Gates P, Talman P (2005). An unusual case of hypertensive encephalopathy. J Clin Neurosci.

[CR22] Sibai BM (1996). Treatment of hypertension in pregnant women. N Engl J Med.

[CR23] Skinhoj E, Strandgaard S (1973). Pathogenesis of hypertensive encephalopathy. Lancet.

[CR24] Sundgren PC, Edvardsson B, Holtås S (2002). Serial investigation of perfusion disturbances and vasogenic oedema in hypertensive encephalopathy by diffusion and perfusion weighted imaging. Neuroradiology.

